# Activation of α7 Nicotinic Acetylcholine Receptor Protects Against 1-Methyl-4-Phenylpyridinium-Induced Astroglial Apoptosis

**DOI:** 10.3389/fncel.2019.00507

**Published:** 2019-11-12

**Authors:** Ye Hua, Beibei Yang, Qiang Chen, Ji Zhang, Jun Hu, Yi Fan

**Affiliations:** ^1^Department of Neurology, The Affiliated Wuxi No. 2 People’s Hospital of Nanjing Medical University, Wuxi, China; ^2^Department of Pharmacology, Neuroprotective Drug Discovery Center, Nanjing Medical University, Nanjing, China; ^3^Division of Clinical Pharmacy, Department of Pharmacy, The First Affiliated Hospital of Nanjing Medical University, Nanjing, China; ^4^Department of Orthopedics, The First Affiliated Hospital of Nanjing Medical University, Nanjing, China

**Keywords:** α7 nicotinic receptor, astrocyte, PNU-282987, apoptosis, 1-methyl-4-phenylpyridinium

## Abstract

Astrocytes, as the largest population of glial subtype, play crucial roles in normal brain function and pathological conditions, such as Parkinson’s disease (PD). Restoring the functions of astrocyte is a promising new therapeutic target for PD. Astrocytes can express multiple types of neurotransmitter receptors, including functional α7 nicotinic acetylcholine receptor (α7nAChR). Previously, we found that a non-selective α7nAChR agonist nicotine exerted a protective effect against H_2_O_2_-induced astrocyte apoptosis *via* an α7nAChR-dependent pathway. However, the molecular mechanism of the antiapoptotic response of astroglial α7nAChR has not been studied. In the present study, using pharmacological inhibition and genetic knockout of α7nAChR, we assessed the antiapoptotic effects of an α7nAChR agonist PNU-282987 in primary cultured astrocytes treated with 1-methyl-4-phenylpyridinium (MPP^+^). PNU-282987 promoted the viability of astrocytes, alleviated MPP^+^ induced apoptosis, and decreased the number of GFAP^+^/TUNEL^+^ cells. Meanwhile, PNU-282987 upregulated the expression of the antiapoptotic protein Bcl-2 and downregulated the expression of the apoptotic protein Bax and cleaved-caspase-3. Moreover, the suppression of the JNK-p53-caspase-3 signaling may underlie the neuroprotective property of PNU-282987. Therefore, PNU-282987 ameliorates astroglial apoptosis induced by MPP^+^ through α7nAChR-JNK-p53 signaling. Our findings suggest that PNU-282987 may be a potential drug for restoring astroglial functions in the treatment of PD.

## Introduction

As the largest population of glial subtype in the central nervous system, astrocytes play crucial roles in maintaining normal brain function and homeostasis (Santello et al., 2019; Valori et al., 2019). Astrocytes provide multiple physiological support functions for neurons, and loss of these critical functions can contribute to the disruption of neuronal function and neurodegeneration (Li et al., 2019; Sorrentino et al., 2019). Besides, astrocytes become activated with heterogeneous and progressive changes in response to pathological conditions, such as inflammatory disease (Iglesias et al., 2017), acute traumatic brain injury (Burda et al., 2016), ischemia/hypoxia (Liu and Chopp, 2016), Alzheimer’s disease (Acosta et al., 2017), and Parkinson’s disease (PD) (Booth et al., 2017). Activated astrocytes leave their quiescent state and are characterized by cellular swelling, proliferation (astrocytosis), and hypertrophy-hyperplasia (astrogliosis) (Pekny and Nilsson, 2005). At the early stage of injury, activated astrocytes are believed to be beneficial to neurons by participating in regulating brain energy metabolism, recycling extracellular ions and neurotransmitter levels, and secreting neuroprotective substances (Efremova et al., 2017; Martin-Jiménez et al., 2017). However, the specific role of prolonged activated astrocytes is still controversial. Activated astrocytes are essential for neuronal survival and functional recovery by the release of neurotrophins (Gozes et al., 1999; Albrecht et al., 2002) and the existence of gap junction (Cotrina et al., 1998; Lin et al., 2010). In contrast, activated astrocytes produce potential toxic mediators and kill neighboring cells in brain injury (Pekny and Nilsson, 2005; Li et al., 2019). Thus, astrocytes act as initiators or contributors in neuropathological conditions *via* both gain-of-function and loss-of-function mechanisms.

As a common neurodegenerative disease, PD is pathologically characterized by the loss of dopaminergic neurons in the substantia nigra pars compacta (SNpc) (Kalia and Lang, 2015). The origin of PD and mechanisms of neuronal degeneration have not yet been fully understood. Compelling evidence certainly indicates that astrocytes have an initiating role in the pathophysiology of PD (Booth et al., 2017). The presence of reactive astrocytes is a crucial aspect of PD pathophysiology in the SNpc (Miklossy et al., 2006). There is also growing evidence that many of the PD-associated genes, such as α-synuclein, DJ-1, ATP13A2, PINK1, and Parkin, are involved in astrocyte-specific functions, including glutamate uptake (Gu et al., 2010; Kim et al., 2016), inflammatory response (Waak et al., 2009; Fellner et al., 2013; Qiao et al., 2016), fatty acid metabolism (Xu et al., 2003; Castagnet et al., 2005), and neurotrophic capacity (Mullett and Hinkle, 2009; Gu et al., 2010; Qiao et al., 2016). Notably, a recent study found that postmortem tissue from PD patients have shown an increase in astrocytic senescence, and removing these senescent cells prevented the symptoms of PD in a mouse model (Chinta et al., 2018). These studies suggest that astrocyte dysfunction plays an essential role in the development and exacerbation of PD, and targeting and restoring the functions of astrocyte are promising new therapeutic targets of neuroprotection.

Alpha7 nicotinic acetylcholine receptor (α7nAChR) is one of the most abundant nAChRs in the mammalian brain (Bertrand et al., 2015). Recent studies suggest that α7nAChR activation may be a crucial mechanism underlying the anti-inflammatory (Egea et al., 2015; Echeverria et al., 2016), antiapoptotic (Kim et al., 2012), and neuroprotective potential of nicotine (Quik et al., 2015; Liu et al., 2017) in several neuropathological conditions. Our previous studies found that α7nAChRs may mediate the protective effects of nicotine *in vivo* in PD model mice (Liu et al., 2015, 2017) and *in vitro* in cultured 1-methyl-4-phenylpyridinium (MPP^+^)-induced SH-SY5Y cells (Xu et al., 2019). The expression of functional α7nAChRs has also been reported to be present in astrocytes (Teaktong et al., 2003; Xu et al., 2019). Given that the impairment of astrocyte functions can critically influence neuronal survival, it is critical to evaluate the potential role of astroglial α7nAChR. Recently, we have demonstrated that nicotine exerted a protective effect against H_2_O_2_-induced astrocyte apoptosis, which was abolished by an α7nAChR-selective antagonist (Liu et al., 2015). Astrocyte apoptosis is believed to contribute to the pathogenesis of chronic neurodegenerative disorders, including PD (Takuma et al., 2004). Therefore, targeting astroglial α7nAChR for their antiapoptotic properties may be necessary for guiding disease-modifying therapies for PD. However, the molecular mechanism of the observed antiapoptotic response of astroglial α7nAChR has not been studied.

In this study, we firstly confirmed *in vitro* the neuroprotective effect of an α7nAChR agonist PNU-282987 in astrocytes treated with 1-methyl-4-phenylpyridinium (MPP^+^, a neurotoxin used in cellular models of PD). Then, we showed that PNU-282987 decreased the number of TUNEL^+^/GFAP^+^ cells and alleviated MPP^+^-induced apoptosis. Meanwhile, PNU-282987 upregulated the expression of the antiapoptotic protein Bcl-2 and downregulated the expression of the apoptotic protein Bax and cleaved caspase-3. Moreover, the suppression of the JNK-p53-caspase-3 signaling may underlie the antiapoptotic property of PNU-282987.

## Materials and Methods

### Reagents

Dulbecco’s modified Eagle’s medium (DMEM) and fetal bovine serum (FBS) were purchased from Gibco Lab (Carlsbad, CA, United States). The tetrazolium salt 3-[4,5-dimethylthiazol-2-yl]-2,5-diphenyltetrazolium bromide (MTT), dimethyl sulfoxide (DMSO), 0.25% trypsin solution, PNU-282987, methyllycaconitine (MLA), Triton X-100, penicillin, and streptomycin were purchased from Sigma-Aldrich (St. Louis, MO, United States). Hoechst 33342 staining was purchased from AAT BioQuest (Montreal, CA, United States). Terminal deoxynucleotidyl transferase dUTP nick end labeling (TUNEL)-staining kit was purchased from Roche Applied Science (South San Francisco, CA, United States). ProLong Gold Antifade with 4′,6-diamidino-2-phenylindole (DAPI) was purchased from Invitrogen (Carlsbad, CA, United States). The lactate dehydrogenase (LDH) cytotoxicity detection kit was purchased from Nanjing Jiancheng Bioengineering Institute (Jiangsu, China). The Annexin-V fluorescein isothiocyanate (FITC) apoptosis detection kit was purchased from BD Biosciences (San Jose, CA, United States). Protease inhibitor cocktail and phosphotransferase inhibitor cocktail were purchased from Roche (Indianapolis, IN, United States).

### Animal

Alpha7-nAChR knockout (KO) mice (C57BL/6J background) were purchased from the Jackson Laboratory (B6.129S7-charna7tm1bay, number 003232; Bar Harbor, ME, United States) (Liu et al., 2017). Wild-type (WT) C57BL/6J mice were purchased from the Animal Core Facility of Nanjing Medical University (Nanjing, Jiangsu, China). All mice were housed in animal facilities under a standardized light-dark cycle and had free access to food and water.

### Cell Culture and Treatment

Primary astrocyte cultures were established from midbrain of 1- to 2-day-old KO and WT mice as previously described (Hua et al., 2019). After washing twice with Dulbecco’s phosphate-buffered saline (DPBS), tissues were digested with 0.25% trypsin solution at 37°C for 20 min and stopped by DMEM supplemented with 10% FBS to avoid overtrypsinization, which can severely damage the cells. After centrifugation at 1,500 rpm at room temperature (RT) for 5 min, the cell pellets were resuspended and cultured at 37°C in DMEM with 10% FBS, 100 mg/ml streptomycin, and 100 U/ml penicillin. The culture medium was replaced twice a week. Since neuronal cultures need a higher nutrient-rich media with neuronal supplements (e.g., B-27), neurons cannot survive in astrocyte growth medium without these neuronal supplements. Astrocytic cultures were subjected to one passage for purification purposes. The purity of astrocyte was more than 95% determined by immunostaining with antibodies against glial fibrillary acidic protein (GFAP). Astrocytes were incubated for 24 h with 800 μM MPP^+^ after preincubation with 0.001∼100 μM PNU-282987 with or without an antagonist of α7nAChR MLA for 30 min.

### MTT Assay

As previously described (Hua et al., 2019), cell viability was determined through the MTT assay. Briefly, cultures of astrocytes were plated on 96-well plates in growth media and were treated following the experimental design. After the supernatants were removed, the cells were incubated with 200 μl of 0.5 mg/ml MTT for 4 h at 37°C in a humidified atmosphere. Then, the MTT medium was removed and replaced with 200 μl DMSO at RT for 30 min. The absorbance of each well was measured at 570 nm with a microplate reader. After background OD subtraction, the results were expressed as a percentage in comparison to the control.

### LDH Release

Using the LDH cytotoxicity detection kit, cell viability was measured by LDH release from mouse astrocytes. According to the manufacturer’s instructions, the samples were quantified at 490 nm with a microplate reader.

### Hoechst 33342 Staining

The apoptosis of astrocytes was detected by the Hoechst 33342 staining. Briefly, cultured astrocytes were fixed with 4% paraformaldehyde in 0.1 M phosphate-buffered saline (PBS) for 15 min and then stained with 5 mg/ml Hoechst 33342 for 15 min. After three rinses with PBS for 5 min, apoptotic cells characterized by nuclear condensation or fragmentation were visualized by fluorescence microscopy (Olympus BX 60, Tokyo, Japan).

### Flow Cytometry Analysis of Apoptosis

The apoptosis of astrocytes was estimated using the Annexin V-FITC apoptosis detection kit according to the manufacturer’s instructions. The samples from cultured astrocytes were incubated for 15 min in the dark with Annexin V-FITC and PI and then analyzed in a Guava easyCyte^TM^ 8HT system (Millipore, Billerica, MA, United States). The number of cells in each category is expressed as the percentage of the total number of cells counted.

### TUNEL Assay

Primary astrocytes were grown on poly-d-lysine precoated glass slides, fixed, and permeabilized in 0.1% Triton X-100 (vol/vol) and 5% (vol/vol) bovine serum albumin (BSA) in PBS for 30 min. Subsequently, the percentage of apoptotic astrocytes was determined by double immunofluorescence staining for TUNEL and GFAP (1:1,000, #ab53554, Abcam, Cambridge, MA, United States) overnight at 4°C. Then, the slides were washed three times with PBS and incubated with the appropriate secondary antibody for 1 h at RT. After incubation with the anti−fade reagent with DAPI, the green fluorescein-labeled DNA was visualized by fluorescence microscopy (Olympus BX 60, Tokyo, Japan).

### Western Blotting

As previously described (Hua et al., 2019), cell pellets were homogenized in 150 μl lysis buffer (50 mM Tris–HCl, pH 7.4, 150 mM NaCl, 1% Nonidet P-40, 1 mM sodium orthovanadate, protease inhibitor cocktail, and phosphotransferase inhibitor cocktail). After being electro-transferred to polyvinylidene fluoride membranes, proteins were incubated in tris-buffered saline with tween (TBST, pH 7.4, 10 mM Tris–HCl, 150 mM NaCl, 0.1% Tween-20) with 5% BSA at RT for 1 h. Then, the following primary antibodies were incubated with: anti-Bax (1:1,000, #2772, Cell Signaling Technology, Danvers, MA, United States), anti-Bcl2 (1:1,000, #2876, Cell Signaling Technology, Danvers, MA, United States), anti-cleaved-caspase-3 (1:500, #9664, Cell Signaling Technology, Danvers, MA, United States), anti- caspase-3 (1:1,000, #9662, Cell Signaling Technology, Danvers, MA, United States), anti-p-p53 (1:500, #9284, Cell Signaling Technology, Danvers, MA, United States), anti-p53 (1:1,000, #2524, Cell Signaling Technology, Danvers, MA, United States), anti-p-JNK (1:1,000, #4668, Cell Signaling Technology, Danvers, MA, United States), anti-JNK (1:1,000, #9252, Cell Signaling Technology, Danvers, MA, United States), and β-actin (BL005B, 1:5,000, Biosharp, Beijing, China) in TBST at 4°C overnight. Followed by four washes, the membranes were incubated for 1 h in TBST containing a secondary antibody. The blots were incubated in HRP-conjugated secondary antibodies, and signals were detected by enhanced chemiluminescence (ECL) reagents (Pierce, Rockford, IL, United States). The membranes were scanned and analyzed using the Tanon 5200 (Tanon, Shanghai, China).

### Statistical Analysis

All data were expressed as mean ± SEM. Differences among means were analyzed using SPSS 17.0 statistical software by means of one-way or two-way analysis of variance (ANOVA), followed by Tukey’s multiple comparison *post hoc* test. In all values, *p* < 0.05 was considered statistically significant.

## Results

### PNU-282987 Reverses MPP^+^-Induced Cytotoxicity in Primary Cultured Astrocytes

**FIGURE 1:**
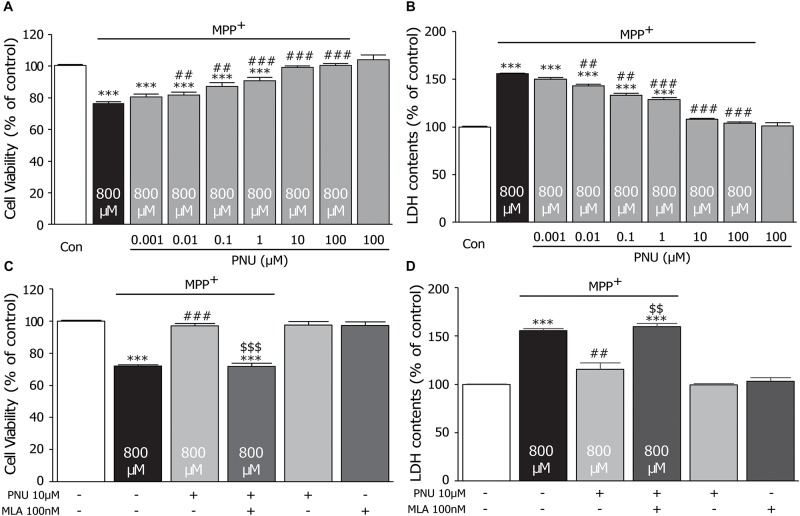
The protective effects of PNU-282987 on cell viability of primary cultured astrocytes after MPP^+^ injury. A 30-min pretreatment with PNU-282987 (0.001, 0.01, 0.1, 1, 10, or 100 μM) inhibited 800 μM MPP^+^-induced cultured astroglial death after 24 h. **(A)** Pretreatment with PNU-282987 significantly promoted the viability of astrocytes in a concentration-dependent manner after MPP^+^ injury. **(B)** Pretreatment with PNU-282987 decreased the release of LDH from astrocytes in a concentration-dependent manner after MPP^+^ injury. **(C)** Pretreatment with 10 μM PNU-282987 with 100 nM MLA abolished the increased viability of astrocytes shown in the pretreatment with PNU-282987 only after MPP^+^ injury. **(D)** Pretreatment with 10 μM PNU-282987 with 100 nM MLA abolished the reduced release of LDH from astrocytes shown in the pretreatment with PNU-282987 only after MPP^+^ injury. Data are presented as mean ± SEM of three independent experiments. ^∗∗∗^*p* < 0.001 vs. control group; ^###^*p* < 0.001, ^##^*p* < 0.01 vs. MPP^+^ treatment group; ^$$$^ *p* < 0.001, ^$$^ *p* < 0.01 vs. PNU-282987 treatment group. Con, control; PNU, PNU-282987; MLA, methyllycaconitine; LDH, lactate dehydrogenase.

**FIGURE 2:**
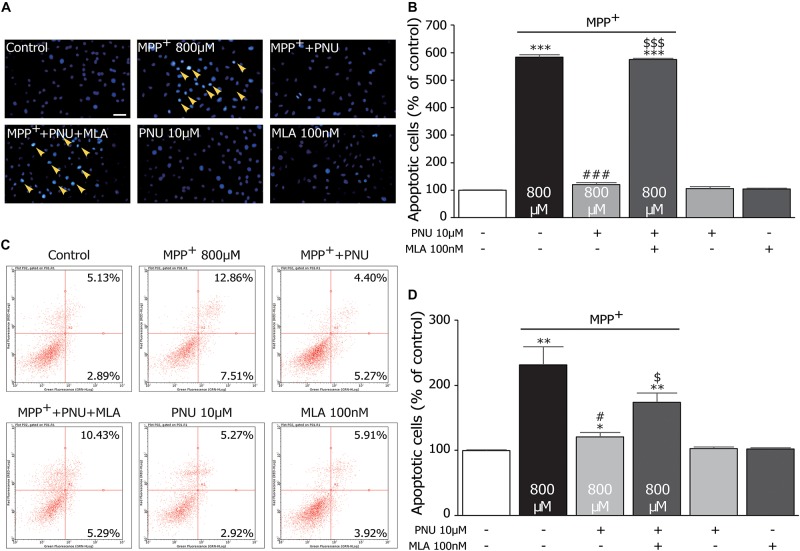
The protective effects of PNU-282987 on MPP^+^-induced apoptosis in primary cultured astrocytes. **(A)** Apoptotic cells characterized by cell shrinkage and chromatin condensation were examined by Hoechst 33342 staining and indicated by a yellow arrow. Scale bar: 200 μm. **(B)** Quantification for staining analysis was presented. **(C)** Apoptosis cells were stained with Annexin-V and propidium iodide and analyzed by flow cytometry. Representative picture and quantification for analysis treatment were shown. **(D)** Quantification for flow cytometry analysis was presented. Data are presented as mean ± SEM of three independent experiments. ^∗∗∗^*p* < 0.001, ^∗∗^*p* < 0.01, and ^∗^*p* < 0.05 vs. control group; ^###^*p* < 0.001 and ^#^*p* < 0.05 vs. MPP^+^ treatment group; ^$$$^*p* < 0.001 and ^$^*p* < 0.05 vs. PNU-282987 treatment group. Con, control; PNU, PNU-282987.

To explore the protective effect of the α7nAChR agonist PNU-282987 on the MPP^+^-induced cell injury, we pretreated cultured astrocytes with different concentrations of PNU-282987 (0.001, 0.01, 0.1, 1, 10, and 100 μM) for 30 min and then incubated with 800 μM MPP^+^ for 24 h, as shown in Figure 1. In cultured astrocytes, 800 μM MPP^+^ caused a 23.3% reduction in control cell viability (100.4% ± 0.6 and 76.7% ± 0.7; control and MPP^+^ alone, respectively; *p* < 0.0001 by one-way ANOVA). PNU-282987 treatment significantly enhanced cell viability in a concentration-dependent manner (80.5% ± 1.7, 81.7% ± 1.9, 87.2% ± 2.2, 90.6% ± 2.2, 99.0% ± 1.0, and 100.4% ± 1.3; 0.001, 0.01, 0.1, 1, 10, and 100 μM of PNU-282987, respectively; *p* = 0.067 for 0.001 μM, *p* = 0.031 for 0.01 μM, *p* = 0.001 for 0.1 μM, *p* < 0.001 for 1∼100 μM by one-way ANOVA) compared with the MPP^+^ only group (Figure 1A). Meanwhile, MPP^+^ alone for 24 h increased LDH leakage by 56.0% compared to the control cells (100.0% ± 0.4 and 156.0% ± 1.7; control and MPP^+^ alone, respectively; *p* < 0.001 by one-way ANOVA), whereas pretreatment with PNU-282987 attenuated this LDH leakage (150.3% ± 1.2, 143.2% ± 2.5, 133.3% ± 2.4, 128.6% ± 1.9, 108.0% ± 3.2, and 103.9% ± 1.7; 0.001, 0.01, 0.1, 1, 10, and 100 μM of PNU-282987, respectively; *p* = 0.050 for 0.001 μM, *p* = 0.014 for 0.01 μM, *p* = 0.002 for 0.1 μM, *p* < 0.001 for 1∼100 μM by one-way ANOVA) in cultured astrocytes (Figure 1B). There was no significant difference in the viability of cells (103.9% ± 3.2) or the leakage of LDH (101.2% ± 1.8) with 100 μM PNU-282987 alone. Furthermore, the neuroprotective effects of PNU-282987 on cell viability (100.0% ± 1.3 in control; 72.1% ± 0.7 in MPP^+^ alone, *p* < 0.001; 96.9% ± 1.8 in MPP^+^ + PNU-282987, *p* < 0.001 vs. MPP^+^ alone; 71.9% ± 1.7 in MPP^+^ + PNU-282987 + MLA, *p* < 0.001 vs. control, *p* < 0.001 vs. MPP^+^ + PNU-282987; one-way ANOVA) and LDH leakage (100.0% ± 0.3 in control; 155.6% ± 2.1 in MPP^+^ alone, *p* < 0.001; 115.5% ± 6.8 in MPP^+^ + PNU-282987, *p* = 0.005 vs. MPP^+^ alone; 159.8% ± 3.3 in MPP^+^ + PNU-282987 + MLA, *p* < 0.001 vs. control, *p* = 0.004 vs. MPP^+^ + PNU-282987; one-way ANOVA) of cultured astrocytes were blocked by 100 nM MLA (Figures 2C,D). MLA alone did not affect cell viability (97.3% ± 2.9) and LDH leakage (103.1% ± 3.7) of cultured astrocytes. These results indicate that PNU-282987 protects against MPP^+^-induced astrocyte damage and also supports the hypothesis that the neuroprotective effects of PNU-282987 on astrocytes are mediated through α7nAChRs. Based on the observations, we chose 10 μM PNU-282987 as the optimal concentration for the following experiment.

### PNU-282987 Alleviates MPP^+^-Induced Apoptosis in Primary Cultured Astrocytes

To further investigate the effects of PNU-282987 on the neurotoxicity of MPP^+^ the MPP^+^-induced apoptotic changes of cultured astrocytes with or without PNU-282987 were investigated, as shown in Figure 2. The Hoechst 33258 staining assay revealed that the rate of apoptotic cells, as indicated by dense granular fluorescence, was increased in MPP^+^ only group compared with the control group (Figures 2A,B; 100.3% ± 1.1 and 583.1% ± 8.8; control and MPP^+^ alone, respectively; *p* < 0.001 by one-way ANOVA). When pretreated with 10 μM PNU-282987 before exposure to 800 μM MPP^+^ for 24 h, the rate of apoptotic cells was significantly reduced (Figures 2A,B; 119.7% ± 8.1 in MPP^+^ + PNU-282987; *p* < 0.001 vs. MPP^+^ alone by one-way ANOVA), revealing that PNU-282987 can protect cultured astrocytes against MPP^+^-induced apoptosis. Similarly, a flow cytometric analysis for apoptosis also indicated that 800 μM MPP^+^ caused significant apoptosis (99.8% ± 1.3 and 231.5% ± 28.0; control and MPP^+^ alone, respectively; *p* = 0.009 by one-way ANOVA), which was inhibited by pretreatment with 10 μM PNU-282987 in cultured astrocytes (Figures 2C,D; 173.7% ± 14.2 in MPP^+^ + PNU-282987; *p* = 0.018 vs. MPP^+^ alone by one-way ANOVA). These antiapoptotic effects of PNU-282987 were expectedly reversed by pretreatment with 100 nM MLA (Figure 2B; 575.2% ± 3.9 in MPP^+^ + PNU-282987 + MLA; *p* < 0.001 vs. MPP^+^ + PNU-282987 by one-way ANOVA; Figure 2D; 173.7% ± 14.2 in MPP^+^ + PNU-282987 + MLA; *p* = 0.028 vs. MPP^+^ + PNU-282987 by one-way ANOVA). These findings confirm that PNU-282987 produces antiapoptotic effects in MPP^+^-stimulated neurotoxicity *via* astroglial α7nAChRs.

### PNU-282987 Decreases the Number of TUNEL^+^/GFAP^+^ Cells in Primary Cultured Astrocytes

**FIGURE 3:**
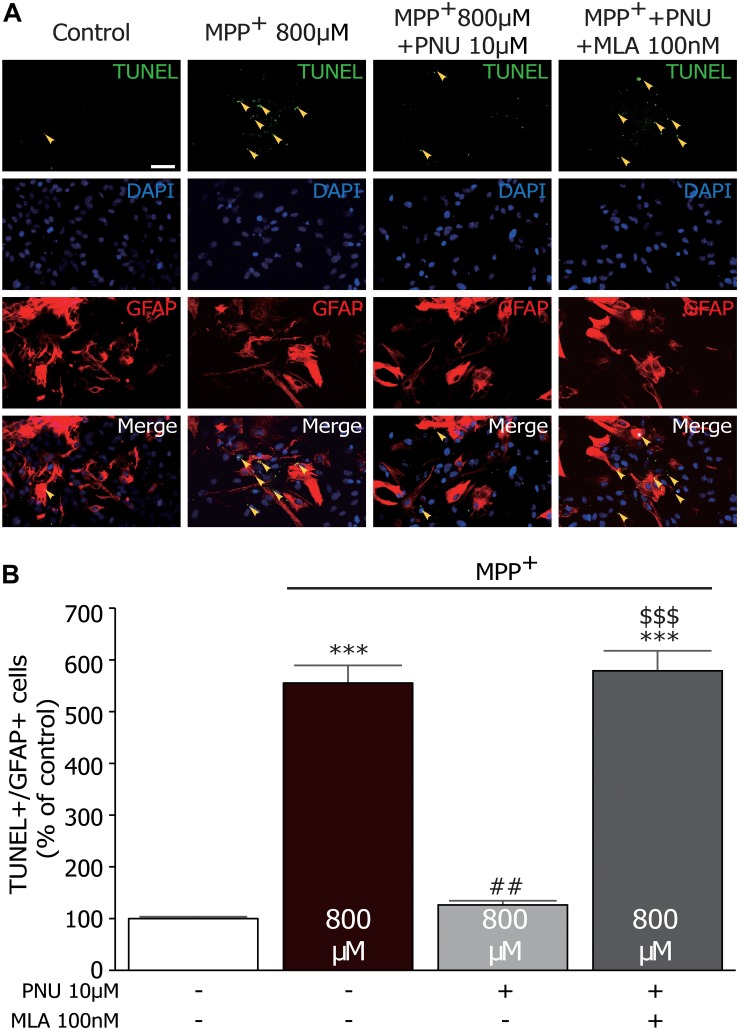
The effects of PNU-282987 on MPP^+^-induced increase of TUNEL^+^/GFAP^+^ cells in primary cultured astrocytes. **(A)** Astrocytes were incubated with primary antibody against GFAP and TUNEL. Images were collected on the fluorescence microscopy. The yellow arrows show significantly increased expression of TUNEL staining in the nucleus of astrocytes. **(B)** Quantitative analysis of PNU-282987-induced protection as measured by TUNEL^+^/GFAP^+^ staining produced a significant neuroprotective effect. ^∗∗∗^*p* < 0.001 vs. control group; ^##^*p* < 0.01 vs. MPP^+^ treatment group; ^$$$^*p* < 0.001 vs. PNU-282987 treatment group. Con, control; PNU, PNU-282987; TUNEL, terminal deoxynucleotidyl transferase dUTP nick end labeling; GFAP, glial fibrillary acidic protein.

To confirm the effect of PNU-282987 on astroglial apoptosis, we examined MPP^+^-stimulated astrocytes using GFAP and TUNEL double labeling. As shown in Figure 3, very few TUNEL^+^ cells were observed in control cultures, whereas substantial TUNEL^+^ cells were detected after 24 h exposure to MPP^+^ (100.2% ± 1.1 and 555.9% ± 40.8; control and MPP^+^ alone, respectively; *p* < 0.001 by one-way ANOVA). Pretreatment with 10 μM PNU-282987 decreased the number of TUNEL^+^/GFAP^+^ cells (127.0% ± 3.9; *p* < 0.001 vs. MPP^+^ alone by one-way ANOVA), which could be blocked by 100 nM MLA (580.2% ± 42.8; *p* < 0.001 vs. MPP^+^ + PNU-282987 by one-way ANOVA). Altogether, these results indicate that α7nAChR activation has been shown to protect astrocytes from MPP^+^-induced apoptosis.

### Pnu-282987 Exerts the Antiapoptotic Effect *via* Suppression of Jnk-p53-Caspase-3 Signaling in Primary Cultured Astrocytes

**FIGURE 4:**
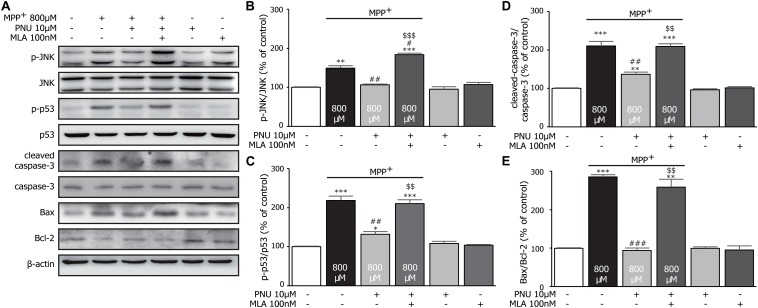
The effects of PNU-282987 on MPP^+^-induced expression of JNK-p53-caspase-3 signaling pathway in primary cultured astrocytes. **(A)** Representative immunoblot of p-JNK, JNK, p-p53, p53, cleaved-caspase-3, caspase-3, Bax, and Bcl-2 in cultured astrocytes. **(B)** Quantitation of p-JNK and JNK levels at baseline and in response to MPP^+^ and PNU-282987 with or without MLA. **(C)** Quantitation of p-p53 and p53 levels at baseline and in response to MPP^+^ and PNU-282987 with or without MLA. **(D)** Quantitation of cleaved-caspase-3 and caspase-3 levels at baseline and in response to MPP^+^ and PNU-282987 with or without MLA. **(E)** Quantitation of Bax and Bcl-2 levels at baseline and in response to MPP^+^ and PNU-282987 with or without MLA. Data are presented as mean ± SEM of three independent experiments. ^∗∗∗^*p* < 0.001, ^∗∗^*p* < 0.01, and ^∗^*p* < 0.05 vs. control group; ^###^*p* < 0.001, ^##^*p* < 0.01, and ^#^*p* < 0.05 vs. MPP^+^ treatment group; ^$$$^*p* < 0.001 and ^$$^*p* < 0.01 vs. PNU-282987 treatment group. Con, control; PNU, PNU-282987; MLA, methyllycaconitine.

To examine the expression levels of apoptosis-associated proteins in cultured astrocytes exposed to MPP^+^, the levels of cleaved-caspase-3, Bax, and Bcl-2 were analyzed by Western blot assay. As shown in Figure 4, MPP^+^ stimulated the expression of cleaved-caspase-3 (100.3% ± 1.1 and 210.1% ± 12.3; control and MPP^+^ alone, respectively; *p* < 0.001 by one-way ANOVA) and the ratio of Bax/Bcl-2 (99.5% ± 1.3 and 284.9% ± 6.1; control and MPP^+^ alone, respectively; *p* < 0.001 by one-way ANOVA) compared to the control group. Pretreatment with 10 μM PNU-282987 reversed the MPP^+^-induced alterations of cleaved-caspase-3 (Figures 4A,D; 136.6% ± 6.0; *p* = 0.006 vs. MPP^+^ alone by one-way ANOVA) and Bax/Bcl-2 (Figures 4A,E; 94.0% ± 6.0; *p* < 0.001 vs. MPP^+^ alone by one-way ANOVA). These results suggest that PNU-282987 reduces the ratios of cleaved-caspase-3/caspase-3 and Bax/Bcl-2, preventing MPP^+^-induced apoptosis.

It is worth noting that JNK pathways have been implicated in many forms of neuronal apoptosis, including dopaminergic neurons in 1-methyl-4-phenyl-1,2,3,6-tetrahydropyridine (MPTP)-induced dopaminergic neurotoxicity (Saporito et al., 2000) and MPP^+^-induced cell death (Choi et al., 1999). Furthermore, the phosphorylation of p53 is critically required for the apoptotic pathway by JNK signaling (Fuchs et al., 1998; Miller et al., 2000; Wang and Friedman, 2000). Thus, we hypothesize that these antiapoptotic effects of PNU-282987 in astrocytes are possibly involved in the regulation of JNK/p53 signaling pathways. Compared to the control group, 800 μM MPP^+^ upregulated the expressions of p-JNK (Figures 4A,B; 100.2% ± 1.2 and 149.0% ± 6.4; control and MPP^+^ alone, respectively; *p* = 0.002 by one-way ANOVA) and p-p53 (Figures 4A,C; 100.3% ± 1.3 and 218.6% ± 11.4; control and MPP^+^ alone, respectively; *p* < 0.001 by one-way ANOVA) after 24 h exposure. Meanwhile, the pretreatment of 10 μM PNU-282987 decreased MPP^+^-induced phosphorylation of JNK (106.2% ± 2.0; *p* = 0.003 vs. MPP^+^ alone by one-way ANOVA) and p53 (132.0% ± 6.8; *p* = 0.003 vs. MPP^+^ alone by one-way ANOVA), which was reversed by 100 nM MLA. Thus, we concluded that PNU-282987 was able to inhibit the phosphorylation of JNK-p53 signaling to prevent MPP^+^-induced astroglial apoptosis.

### Deficiency of α7nAChR Diminishes the Antiapoptotic Effects of PNU-282987

**FIGURE 5:**
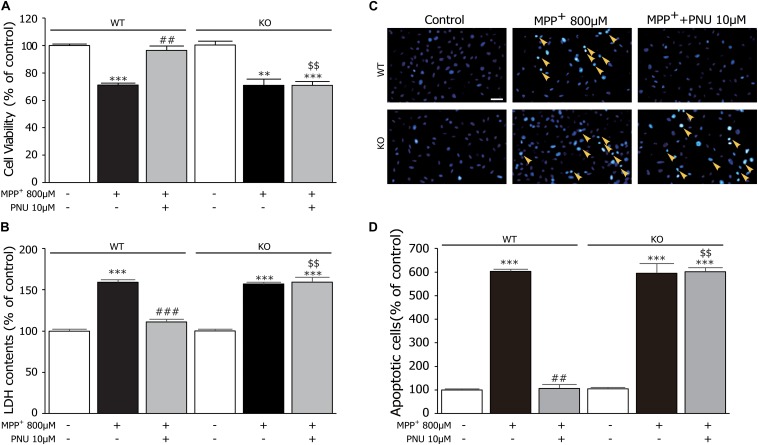
α7nAChR deficiency abrogates the protective effects of PNU-282987 on primary cultured astrocytes after MPP^+^ injury. **(A)** The effects of PNU-282987 on the viability of cells were abolished in cultured astrocytes from KO mice. **(B)** The effects of PNU-282987 on the release of LDH were abolished in cultured astrocytes from KO mice. **(C)** Apoptotic cells characterized by cell shrinkage and chromatin condensation were examined by Hoechst 33342 staining and indicated by a yellow arrow. Scale bar: 200 μm. **(D)** Quantification for Hoechst 33342 staining analysis was presented. Data are presented as mean ± SEM of three independent experiments. ^∗∗∗^*p* < 0.001 and ^∗∗^*p* < 0.01 vs. corresponding control group; ^###^*p* < 0.001 and ^##^*p* < 0.01 vs. corresponding MPP^+^ treatment group; ^$$^*p* < 0.01 vs. PNU-282987-treated WT group. WT, astrocyte from wild-type mice; KO, astrocytes from α7nAChR KO mice; PNU, PNU-282987; LDH, lactate dehydrogenase.

Furthermore, we used primary astrocytes derived from KO mice to investigate the roles of α7nAChR in regulating the antiapoptotic effects of PNU-282987 after MPP^+^ exposure. As shown in Figures 5A,B, two-way ANOVA revealed a highly significant difference in cell viability and LDH leakage by genotype (cell viability: *F*_1_,_17_ = 19.825, *p* = 0.001; LDH leakage: *F*_1_,_17_ = 45.246, *p* < 0.001), treatment (cell viability: *F*_2_,_17_ = 74.184, *p* < 0.001; LDH leakage: *F*_2_,_17_ = 293.169, *p* < 0.001), and genotype × treatment (cell viability: *F*_2_,_17_ = 17.137, *p* < 0.001; LDH leakage: *F*_2_,_17_ = 51.483, *p* < 0.001). As found in the *in vivo* PD animal model (Liu et al., 2017) and *in vitro* SH-SY5Y cells (Xu et al., 2019), α7nAChR deficiency/knockdown did not affect the neurotoxicity of MPP^+^ both in cell viability (Figure 5A; WT: 100.6% ± 0.8 in control and 75.0% ± 1.6 in MPP^+^ alone, KO: 100.4% ± 2.2 in control and 74.1% ± 2.3 in MPP^+^ alone) and LDH leakage (Figure 5B; WT: 100.4% ± 1.1 in control and 155.6% ± 2.1 in MPP^+^ alone, KO: 99.5% ± 0.8 in control and 154.9% ± 1.7 in MPP^+^ alone). However, compared to astrocytes derived from WT mice, the neuroprotective effects of PNU-282987 on cell viability (WT: 96.3% ± 3.6; KO: 74.0% ± 1.2, *p* < 0.001) and LDH leakage (WT: 116.2% ± 2.6; KO: 156.1% ± 4.1, *p* < 0.001) were abolished in astrocytes derived from KO mice. In addition, the antiapoptotic effect of PNU-282987 confirmed by a decrease in the number of apoptotic nuclei was also eliminated in astrocytes derived from KO mice (Figures 5C,D). These results indicate that the antiapoptotic effect of PNU-282987 in cultured astrocytes stimulated by MPP^+^ is *via* an α7nAChR-dependent mechanism.

### Deficiency of α7nAChR Abolishes the Inhibitory Effect of PNU-282987 on JNK-p53-Caspase-3 Signaling

**FIGURE 6:**
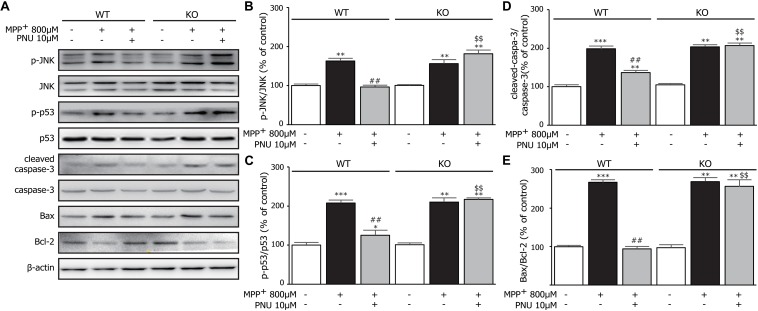
The effects of α7nAChR deficiency on MPP^+^-induced expression of the JNK-p53-caspase-3 signaling pathway in primary cultured astrocytes. **(A)** Representative immunoblot of p-JNK, JNK, p-p53, p53, cleaved-caspase-3, caspase-3, Bax, and Bcl-2 in WT and KO cultured astrocytes. **(B)** Quantitation of p-JNK and JNK levels at baseline and in response to MPP^+^ and PNU-282987. **(C)** Quantitation of p-p53 and p53 levels at baseline and in response to MPP^+^ and PNU-282987. **(D)** Quantitation of cleaved-caspase-3 and caspase-3 levels at baseline and in response to MPP^+^ and PNU-282987. **(E)** Quantitation of Bax and Bcl-2 levels at baseline and in response to MPP^+^ and PNU-282987. Data are presented as mean ± SEM of three independent experiments. ^∗∗∗^*p* < 0.001, ^∗∗^*p* < 0.01, and ^∗^*p* < 0.05 vs. corresponding control group; ^##^*p* < 0.01 vs. corresponding MPP^+^ treatment group; ^$$^*p* < 0.01 vs. PNU-282987-treated WT group. WT, astrocyte from wild-type mice; KO, astrocytes from α7nAChR KO mice; PNU, PNU-282987.

Next, we tested whether α7nAChR deficiency changed the JNK-p53-caspase-3 signaling pathway in MPP^+^-induced astroglial apoptosis. As shown in Figure 6A, two-way ANOVA revealed a highly significant difference in the expressions of p-JNK (genotype: *F*_1_,_17_ = 69.269, *p* < 0.001; treatment: *F*_2_,_17_ = 162.551, *p* < 0.001; genotype × treatment: *F*_2_,_17_ = 77.330, *p* < 0.001), p-p53 (genotype: *F*_1_,_17_ = 42.936, *p* < 0.001; treatment: *F*_2_,_17_ = 227.694, *p* < 0.001; genotype × treatment: *F*_2_,_17_ = 44.312, *p* < 0.001), leaved-caspase-3 (genotype: *F*_1_,_17_ = 85.225, *p* < 0.001; treatment: *F*_2_,_17_ = 503.821, *p* < 0.001; genotype × treatment: *F*_2_,_17_ = 84.738, *p* < 0.001), and the ratio of Bax/Bcl-2 (genotype: *F*_1_,_17_ = 105.085, *p* < 0.001; treatment: *F*_2_,_17_ = 282.153, *p* < 0.001; genotype × treatment: *F*_2_,_17_ = 90.586, *p* < 0.001). After 24 h exposure to MPP^+^, both KO astrocytes and WT astrocytes exhibited the same alterations in the phosphorylation of JNK and p53, the increased expression of cleaved-caspase-3, and the upregulated ratio of Bax/Bcl-2 (Figure 6). However, α7nAChR deficiency diminished the inhibitory effect of PNU-282987 on the phosphorylation of JNK and p53. As expected, α7nAChR deficiency also abolished the regulation of PNU-282987 on the expressions of caspase-3/Bax/Bcl-2 signaling pathways. These data indicated that PNU-282987 alleviated apoptotic cell death induced by MPP^+^ in cultured astrocytes mainly *via* the α7nAChR-JNK-p53 pathway.

## Discussion

The neuroprotective effects of activating α7nAChR on neurodegenerative disorders are receiving increasing attention. Previous studies suggest that α7nAChR activation exerts neuroprotective actions in PD models *in vitro* (Quik et al., 2015) and *in vivo* (Liu et al., 2015, 2017; Quik et al., 2015). We also confirmed that α7-nAChR agonists protected against MPP^+^-induced apoptosis in SH-SY5Y cells *via* activating the ERK/p53 signaling pathway (Xu et al., 2019). In the present study, we further found the antiapoptotic effect of PNU-282987 *in vitro* using primary astrocytes exposed to MPP^+^. PNU-282987 reversed the reduction of cell viability and the increase of cell apoptosis in primary astrocytes after MPP^+^ exposure. Using α7nAChR KO mice, we indicate that the underlying protective mechanism of PNU-282987 was mediated through activating α7nAChR, suppressing the phosphorylation of JNK and p53, and then regulating the caspase-3/Bax/Bcl-2 signaling pathway. Therefore, the antiapoptotic effect of PNU-282987 on astrocytes may offer therapeutic benefits for neurodegenerative disorders such as PD.

MPP^+^ is the toxic metabolite of MPTP and is formed by the oxidation of monoamine oxidase B, which is predominantly located in the astrocyte (Ransom et al., 1987). After taking up the dopamine reuptake system, MPP^+^ can inhibit the function of mitochondrial complex I and subsequently induce selective dopaminergic neuronal degeneration in the substantia nigra (Langston et al., 1984). MPP^+^ also has a direct toxic effect on astrocytes and results in the impairment of astrocyte functions, such as glutamate homeostasis (Di Monte et al., 1999), mitochondrial damage (Di Monte et al., 1992), oxidative stress (Wong et al., 1999), and apoptosis (Zhang et al., 2007). In agreement with these previous studies, we also found in the present study that treating the primary astrocytes with MPP^+^ induced a loss of cell viability, cell shrinkage, and caspase-3 activation, which was associated with the increased Bax/Bcl-2 ratio. Furthermore, *via* a pharmacologic approach (using a selective α7nAChR inhibitor) and a genomic strategy (using α7nAChR KO mice), we characterized the functional role of α7nAChR in astrocytes and found that PNU-282987 attenuated MPP^+^-induced astroglial apoptosis mainly *via* the α7nAChR-JNK-p53 pathway. The protective effect of PNU-282987 on astroglial survival may have a beneficial effect on the maintenance of astroglial function and attenuate delayed neuronal death in PD. Notably, previous studies indicate no vulnerability to MPTP/MPP^+^ in α7nAChR KO mice (Liu et al., 2017) or knockdown SH-SY5Y cell (Xu et al., 2019). The findings in this study also showed no significant effect of α7nAChR deficiency on MPP^+^ toxicity in cultured astrocytes, with no observed difference in alterations in cell apoptosis and JNK-p53-caspase-3 signaling. These data suggest that the expression of α7nAChR may not be necessary for MPTP/MPP^+^-induced neurotoxicity both in neurons and astrocytes.

Increasing evidence reveals that apoptotic cell death serves an essential role in the pathogenesis of PD, causing the death of dopamine neurons in the substantia nigra (Burke, 1998). Astrocyte apoptosis is identified after reactive astrocytosis and contributes to the pathogenesis of brain injuries, including cerebral ischemia, Alzheimer’s disease, and PD (Takuma et al., 2004). Several signals, such as glutamate toxicity, cytosolic Ca^2+^ elevation, oxidative stress, mitochondrial dysfunction, and inflammatory injury, can induce apoptosis in astrocytes *in vivo* and *in vitro*. As one of the major signaling cassettes of the mitogen-activated protein kinase (MAPK) signaling pathway, JNK signaling is a known important mediator of MPTP/MPP^+^-induced apoptosis (Choi et al., 1999; Saporito et al., 2000). Additionally, numerous studies reported the ability of JNK to bind to and phosphorylate p53 (Fuchs et al., 1998; Wang and Friedman, 2000). During stress, the JNK signaling pathway can increase p53 transactivation and phosphorylation and then potentiate p53-dependent apoptosis (Miller et al., 2000). In the present study, we found that MPP^+^ induced an increase in the cellular levels of p-JNK, p-p53, and cleaved caspase-3. Moreover, pharmacologic inhibition or genetic KO of α7nAChR diminished the inhibitory effects of PNU-282987 on MPP^+^-induced activation of JNK, p53, and caspase-3. Thus, it is reasonable to indicate that α7nAChR activation prevents MPP^+^-induced astroglial apoptosis *via* the inhibition of the JNK-p53-pathway.

Our previous study showed the regulative effects of PNU−282987 on the phosphorylation of JNK in SH−SY5Y cells *via* α7nAChRs−independent signaling (Xu et al., 2019). Notably, these inhibitory effects can be reversed by antagonism of α7nAChR but not by knockdown of α7−nAChR. Indeed, gene KO of α7nAChR involves the deletion strategy targeted exons 8–10 (Orr-Urtreger et al., 1997). KO mice show an absence of high-affinity [I-125] alpha-bungarotoxin sites, although no detectable abnormalities of high-affinity nicotine binding sites. However, gene knockdown leads to the degradation of that mRNA and abortive protein translation only. The expression of functional α7−nAChR was still detectable in α7−siRNA−transfected SH−SY5Y cells. In addition, there are lots of differences between adult and newborn rodents, such as GABA-dependent action potentials (Wang et al., 2001). The JNK and p53 pathways may play distinct roles in neonatal and adult astrocytes, and adult astrocyte cultures may be more useful in the studies of PD. Therefore, considering astrocyte cultures from newborn mice in our present study, further experiments using conditional KO mice of α7−nAChR or adult astrocyte cultures are needed to examine how PNU−282987 modulates the JNK-p53 pathway.

## Conclusion

In the present study, using either pharmacological inhibition or genetic KO of α7nAChR, we found that PNU−282987 protected against the MPP^+^−induced apoptosis of cultured astrocytes *via* α7nAChR-JNK-p53 signaling pathway. These findings indicate the important roles of JNK-p53 signaling in the antiapoptotic response of astroglial α7nAChR and suggest that α7nAChR agonists may be validated as a potential target for modulating astrocyte activity to treat PD.

## Data Availability Statement

The data that support the findings of this study are available from the corresponding author upon reasonable request.

## Ethics Statement

The animal study was reviewed and approved by the Institutional Animal Care and Use Committee of Nanjing Medical University. Written informed consent was obtained from the owners for the participation of their animals in this study.

## Author Contributions

YH, JH, and YF conceived and planned the experiments. YH and BY carried out the cellular experiments and performed the biochemical analysis. QC and JZ designed and performed the animal experiments. YH and JZ contributed to the interpretation of the results. YH and YF wrote the manuscript. All the authors reviewed and approved the final manuscript.

## Conflict of Interest

The authors declare that the research was conducted in the absence of any commercial or financial relationships that could be construed as a potential conflict of interest.
